# The Heterologous Expression of a *Chrysanthemum nankingense* TCP Transcription Factor Blocks Cell Division in Yeast and *Arabidopsis thaliana*

**DOI:** 10.3390/ijms20194848

**Published:** 2019-09-29

**Authors:** Xiangyu Qi, Yixin Qu, Ri Gao, Jiafu Jiang, Weimin Fang, Zhiyong Guan, Fei Zhang, Shuang Zhao, Sumei Chen, Fadi Chen, Haibin Wang

**Affiliations:** College of Horticulture, Nanjing Agricultural University, Key laboratory of Landscape Agriculture, Ministry of Agriculture, Nanjing 210095, China

**Keywords:** transcription profiling, fission yeast, cell proliferation, promoter

## Abstract

Both the presence of, and the important contribution to growth and development made by TCP transcription factors, have been established in various plant species. Here, a *TCP4* homolog isolated from *Chrysanthemum nankingense* was shown to be more strongly transcribed in the diploid than in the autotetraploid form of the species. *CnTCP4* was shown to encode a member of the class II TCP family and to be transcribed most strongly in the leaf and ligulate flowers. Its transcription was found to be substantially inhibited by spraying the plant with the synthetic cytokinin 6-benzylaminopurine. The transient expression of *CnTCP4* in onion epidermal cells showed that its product localized to the nucleus, and a yeast one hybrid assay suggested that its product had transcriptional activation ability. The constitutive expression of *CnTCP4* in fission yeast suppressed cell proliferation, inducing the formation of longer and a higher frequency of multinuclated cells. Its constitutive expression in *Arabidopsis thaliana* reduced the size of the leaves. The presence of the transgene altered the transcription of a number of cell division-related genes. A yeast one hybrid assay identified a second *TCP* gene (*CnTCP2*) able to interact with the *CnTCP4* promoter. A transient expression experiment in *Nicotiana benthamiana* leaves showed that CnTCP2 was able to activate the *CnTCP4* promoter. Like *CnTCP4, CnTCP2* was shown to encode a member of the class II TCP family, to be transcribed most strongly in the leaf and ligulate flowers, and to be suppressed by exogenous 6-benzylaminopurine treatment. The CnTCP2 protein also localized to the nucleus, but had no transcriptional activation ability. Its constitutive expression in *A. thaliana* had similar phenotypic consequences to those induced by *CnTCP4*.

## 1. Introduction

Development in a multicellular organism requires precise control over cell proliferation and expansion. The various phases of the cell cycle each depend largely on the activity of cyclins and cyclin-dependent kinases (CDKs) [1], with a contribution in plants also being made by members of the TCP transcription factor family [2,3,4]. The characteristic feature of the TCPs is a 59 residue stretch, which forms the basic helix-loop-helix (bHLH) motif central to the proteins’ ability to bind to DNA and to direct protein–protein interactions [5]. Both the *Arabidopsis thaliana* and rice genomes harbor over 20 genes encoding a TCP [6]. Based on variation in the TCP domain sequence, the *TCP*s have been divided into two distinct classes, referred to as class I/PCF/TCP-P and class II/TCP-C [7,8]. While the former act to promote cell division, the latter repress it [6,9]. The products of the *A. thaliana* class I genes *AtTCP14* and *AtTCP15* both drive cell division in young internodes [10], while the consequence of knocking out the class I genes *AtTCP9* and *AtTCP20* is to inhibit cell division and to promote cell expansion [11]. The product of the class II gene *AtTCP4* represses cell proliferation in the *A. thaliana* leaf [12]. AtTCP4 activates *AtGIS,* so reducing its presence results in an increase in trichome branching [3].

The diploid species *Chrysanthemum nankingense* is a near relative of the commercially important ornamental species *C. morifolium* [13,14]. The product of the TCP I gene *DgBRC1* represses the formation of lateral branching [15], that of *CmCYC* regulates the growth of ray florets [16], while that of the gene *CmTCP14* suppresses the size of the organ [17]. As yet, no attempt has been made to characterize the complement of *TCP* genes harbored by the *C. nankingense* genome. An analysis of an autotetraploid *C nankingense* showed that its leaves were larger than those of its diploid progenitor [18]. The experiments reported here were designed to reveal the transcriptional behavior of *CnTCP4* in both diploid and autotetraploid forms of the species, and to study the phenotypic effect of heterologously expressing it in both fission yeast and *A. thaliana*.

## 2. Results

### 2.1. Characterization of a TCP4 Homolog in C. nankingense

**Figure 1:**
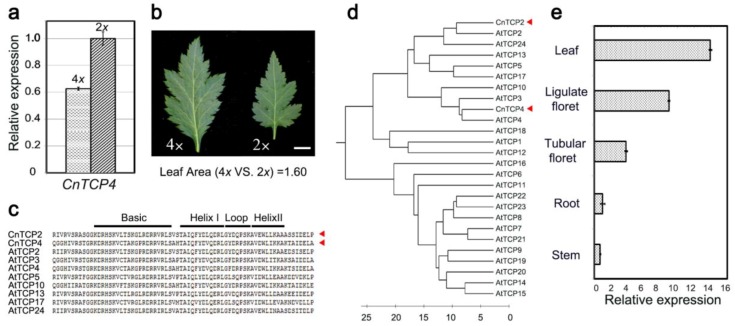
The *C. nankingense* homolog of *AtTCP4.* (**a**) *CnTCP4* was transcribed more abundantly in the diploid than in the autotetraploid form of *C. nankingense*. Values shown as mean ± SE (*n =* 3). (**b**) The leaf of the autotetraploid was larger than that of the diploid. Bar: 1 cm. (**c**) Alignment of the polypeptide sequences of CnTCP4, CnTCP2, and *A. thaliana* class II TCPs. The key domains were identified. (**d**) The phylogenetic relationship of CnTCP4 and CnTCP2 to the family of *A. thaliana* TCPs. (**e**) The transcription of *CnTCP4* throughout the *C. nankingense* plant. Values shown as mean ± SE (*n =* 3).

**Figure 2:**
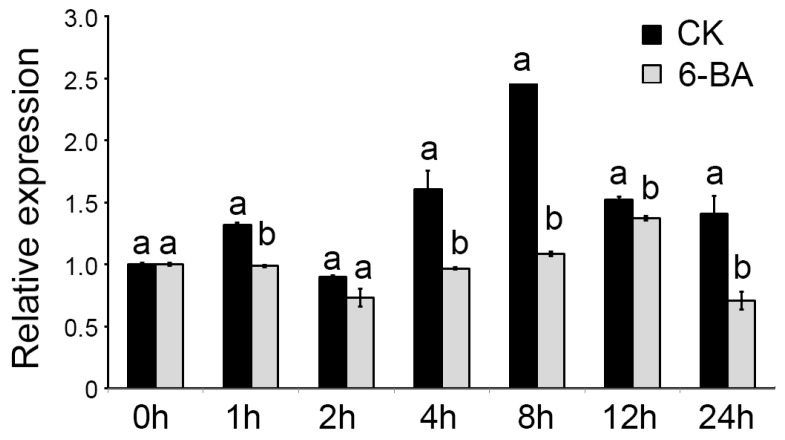
Effects of 6-benzylaminopurine on *CnTCP4* transcription. Values shown as mean ± SE (*n =* 3). Columns headed by a different letter denote transcript abundances differing significantly (*p* < 0.05) from the transcript abundance measured in unsprayed (CK) plants. The *x* axis indicated the time point of the assay following the spray treatment.

The length of the cloned full cDNA sequence was 1812 nt, of which 1203 nt represented the coding sequence. CnTCP4 was therefore predicted to be a 400 residue protein, which included an atypical basic-helix-loop-helix domain (Figure 1c). The sequence was related to the class II set of TCP proteins, as its most closely related sequence was the product of *AtTCP4* (Figure 1d)*. CnTCP4* transcript was abundant in both the leaf and ligulate florets of *C. nankingense* (Figure 1e). A qRT-PCR experiment revealed that the abundance of *CnTCP4* transcript was greater in the leaf of the diploid form of *C. nankingense* than in the leaf of the autotetraploid form (Figure 1a). The autetraploid plants, on the other hand, produced larger leaves than the diploid plants (Figure 1b). *CnTCP4* was significantly downregulated when plants were sprayed with the synthetic cytokinin 6-benzylaminopurine; the abundance of its transcript decreased marginally over the first 4 h following treatment, but fell by at least twofold over the period 8–24 h post treatment (Figure 2).

**Figure 3:**
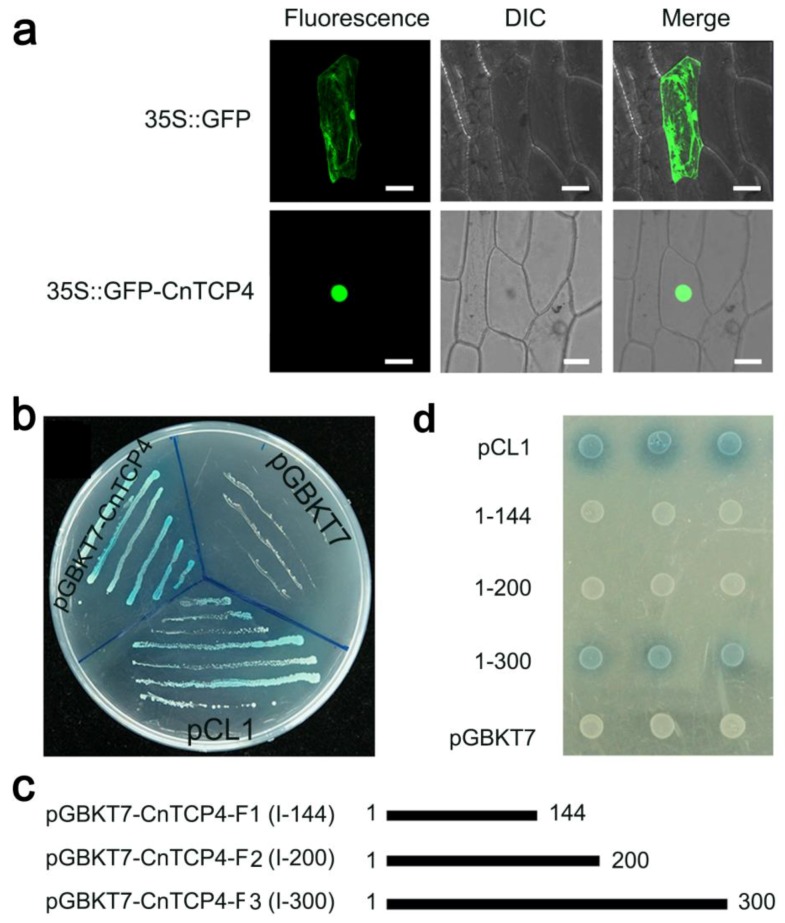
Subcellular localization of CnTCP4 in transgenic onion epidermal cells and the transcriptional activation activity of CnTCP4. (**a**) GFP activity generated by the p35S*::GFP-CnTCP4* transgene introduced into onion epidermis peels. Fluorescence: image recovered from the green fluorescence channel, DIC: image recovered under bright light, merged: an overlay of the fluorescence and DIC images. Bar: 50 μm. (**b**) a yeast one hybrid assay used to detect the transcriptional activation activity of CnTCP4. (**c**) fragment constructs of different C’-deletion of CnTCP4 amino acid. (**d**) transcriptional activation activity of different C’-deletion of CnTCP4.

When the *p35S::GFP-CnTCP4* fusion construct was transiently expressed in onion epidermal cells, the ensuing GFP signal was concentrated in the nucleus, while the introduction of a *p35S::GFP* transgene resulted in a cell-wide distribution of GFP activity (Figure 3a). The upshot of a yeast one hybrid experiment intended to reveal the gene’s transcriptional activity was that yeast cells harboring either pCL1, pGBKT7-*CnTCP4,* or pGBKT7-*CnTCP4-F3* were all able to grow on SD/-His/-Ade and to metabolize X-α-Gal, while cells harboring either the negative control plasmid pGBKT7, pGBKT7-*CnTCP4-F1,* or pGBKT7-*CnTCP4-F2* all failed to show any galactosidase activity (Figure 3b,d). The conclusion was that the full length *CnTCP4* was transcriptionally active, and that residues 201–300 were required for its transcriptional activation.

### 2.2. The Heterologous Expression of CnTCP4 Affected Cell Division of Fission Yeast

**Figure 4:**
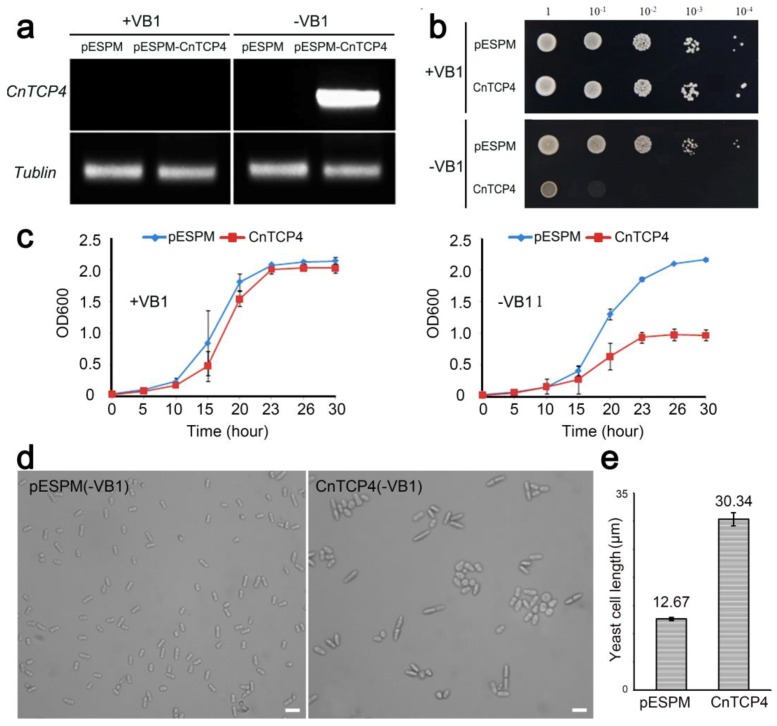
The effect of expressing *CnTCP4* on the growth of fission yeast. (**a**) RT-PCR analysis of the transcription of the *CnTCP4* transgene in yeast. (**b**) Dilution analysis of the yeast transformants harboring *CnTCP4* or an empty vector with (+VB1) or without VB1 (-VB1) on EMM medium. (**c**) The growth of *CnTCP4* and empty transgenic cells in the presence and absence of VB1 in the growth medium. Values shown as mean ±SE *(n* = 3). (**d**) The appearance of cells harboring *CnTCP*4 or an empty vector in response to the absence of VB1 in the medium. Bar: 10 µm. (**e**) The length of cells grown in a medium lacking VB1. Values shown as mean ± SE *(n* = 1000).

**Figure 5:**
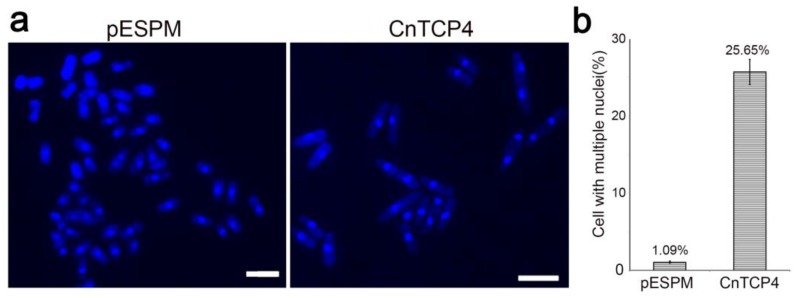
The effect of expressing *CnTCP4* on the appearance of fission yeast grown in a medium lacking VB1. (**a**) DAPI stained cells carrying an empty vector (pESPM) and those carrying the pESPM*::CnTCP4* transgene (CnTCP4). Bar: 10 µm. (**b**) The frequency of cells carrying multiple nuclei. Values shown as mean ± SE (*n* = 1000).

Yeast is used as a model system for studying gene function [19,20], and AtTCP4 protein has been studied in yeast cells [2]. So we investigated the function of CnTCP4 gene using the yeast system. *CnTCP4,* when driven by the *NMT1* promoter, was induced in a medium lacking thiamine (Figure 4a). Under inductive conditions, the proliferation of cells heterologously expressing *CnTCP4* was strongly inhibited, while in a noninductive medium, transformants harboring either *CnTCP4* or an empty pESPM plasmid both grew freely (Figure 4b,c). When cultured under inductive conditions, the length of cells harboring the *CnTCP4* transgene was some 2.5 fold greater than that of those harboring the empty vector (Figure 4d,e), and some of those expressing *CnTCP4* were multiseptated. Inspection of DAPI stained transformed cells revealed morphological differences between those expressing *CnTCP4* and those harboring the empty vector (Figure 5a): the former produced many cells harboring two nuclei or multiple nuclei (Figure 5b). The conclusion was that the expression of *CnTCP4* suppressed cell proliferation via its effect on both cell division and cell expansion.

### 2.3. The Effect of Constitutively Expressing CnTCP4 in A. thaliana

**Figure 6:**
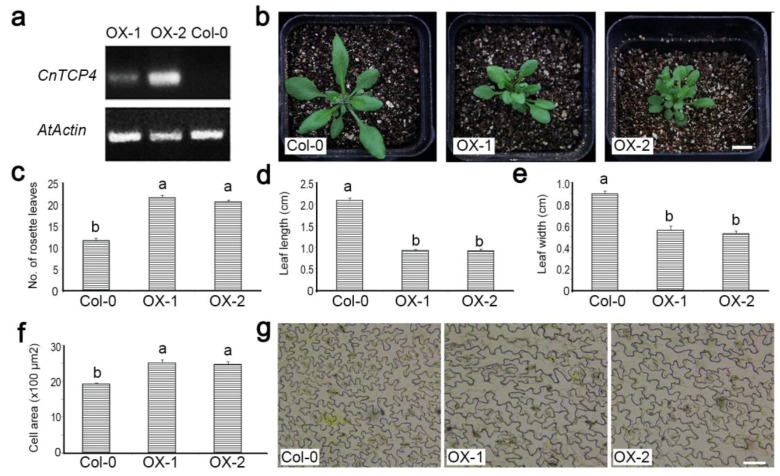
The phenotypic effect of constitutively expressing *CnTCP4* in *A. thaliana*. (**a**) RT-PCR-based identification of the transgenic *A. thaliana* plants OX-1 and OX-2. (**b**) The appearance of 35-day-old wild-type Col-0, OX-1, and OX-2 plants. Bar: 1 cm. Quantification of leaf growth. (**c**) number of rosette leaves (**d**) length of the fifth leaf. (**e**) width of the fifth leaf. (**f**) surface area of cells in the fifth leaf. Values in (**c**) through (**f**) shown as mean ± SE (*n* = 10). (**g**) The appearance of epidermal cells sampled from the fifth leaf. Bar: 50 µm.

**Figure 7:**
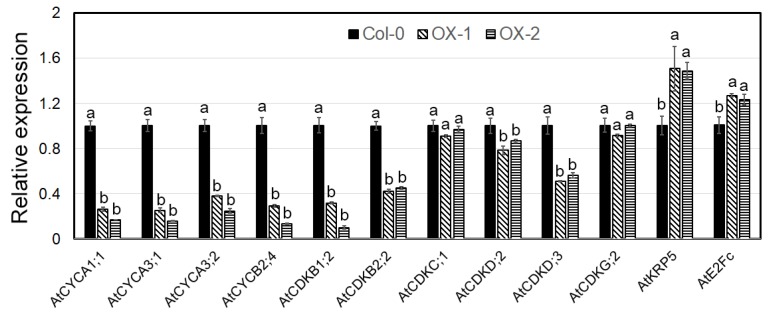
The effect of constitutively expressing *CnTCP4* in *A. thaliana* on the transcription of cell cycle marker genes. Col-0: wild-type plants; OX-1, OX-2: transgenic plants. Values shown as mean ± SE (*n* = 3). Columns headed by a different letter denote transcript abundances differing significantly (*p* < 0.05) from the transcript abundance measured in Col-0 plants.

Two independent constitutive expressors of *CnTCP4* in *A. thaliana* were selected among the T_3_ progeny bred from the original transformed Col-0 material (Figure 6a). Their vegetative growth was severely compromised compared to wild-type plants (Figure 6b); although they formed a higher number of rosette leaves (Figure 6c), both their leaf length (Figure 6d) and width (Figure 6e) were reduced. The size of the cells formed in the epidermis of the lower leaf surface was larger than in wild-type plants (Figure 6f,g). The effect of the transgene on the transcription of a range of cell cycle marker genes was summarized in Figure 7. The abundance of *CYCA1;1*, *CYCA3;1*, *CYCA3;2*, *CYCB2;4*, *CDKB1;2*, *CDKB2;2*, *CDKD;2,* and *CDKD;3* transcripts were lower in the transgenic plants than in wild type, and vice versa for *KRP5* and *E2Fc*, there was no effect on the transcript abundance of either *CDKC;1* or *CDKG;2*.

### 2.4. CnTCP2 Interacted with the CnTCP4 Promoter

**Figure 8:**
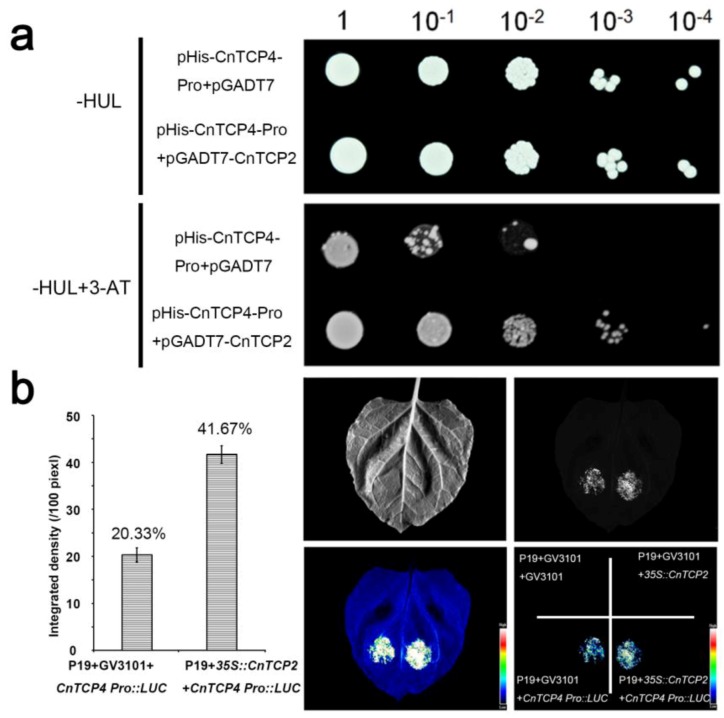
The *CnTCP4* promoter interacted with CnTCP2. (**a**) Yeast one hybrid assay. Dilution analysis of the yeast transformants harboring *CnTCP4-Pro* and empty pGADT7 vector or *CnTCP4-Pro* and pGADT7-CnTCP2 on medium lacking His, Ura, and Leu with 3-AT or without it. -HUL: medium lacking His, Ura, and Leu; (**b**) CnTCP2 activated the *CnTCP4* promoter in *N. benthamiana* leaves. P19+GV3101+GV3101: cultures of P19 plus GV3101 at a ratio of 1:2, P19+GV3101+35S::CnTCP2: cultures of P19 plus GV3101 and p*35S::CnTCP2* at a ratio of 1:1:1, P19+GV3101+CnTCP4 Pro:LUC: cultures of P19 plus GV3101 and pCAMBIA1381Z-Luc-*CnTCP4-*Promoter at a ratio of 1:1:1, P19+35S::CnTCP2+CnTCP4 Pro:LUC: cultures of P19 plus p*35S::CnTCP2* and pCAMBIA1381Z-Luc-*CnTCP4-*Promoter at a ratio of 1:1:1. Values shown as mean ± SE (*n* = 10).

Multiple potential TCP binding sites were identified within the *CnTCP4* promoter fragment (Figure S1), and various ethylene-responsive, gibberellin-responsive, and light-responsive elements were predicted by PLACE software (Table 1). When the *CnTCP4* promoter fragment was subjected to a yeast one hybrid assay, a sequence designated *CnTCP2* was identified as a putative interacting transcription factor. The interaction was confirmed by testing the *CnTCP2* ORF sequence in an independent yeast one hybrid assay featuring the same *CnTCP4* promoter fragment (Figure 8a). In addition, a transient assay conducted in *N. benthamiana* leaves showed that leaves infiltrated with a p*CnTCP4*::*LUC* transgene generated less LUC activity than did leaves co-infiltrated with both p*CnTCP4*::*LUC* and p*35S::CnTCP2* (Figure 8b).

### 2.5. Characterization of the CnTCP2 Sequence

**Figure 9:**
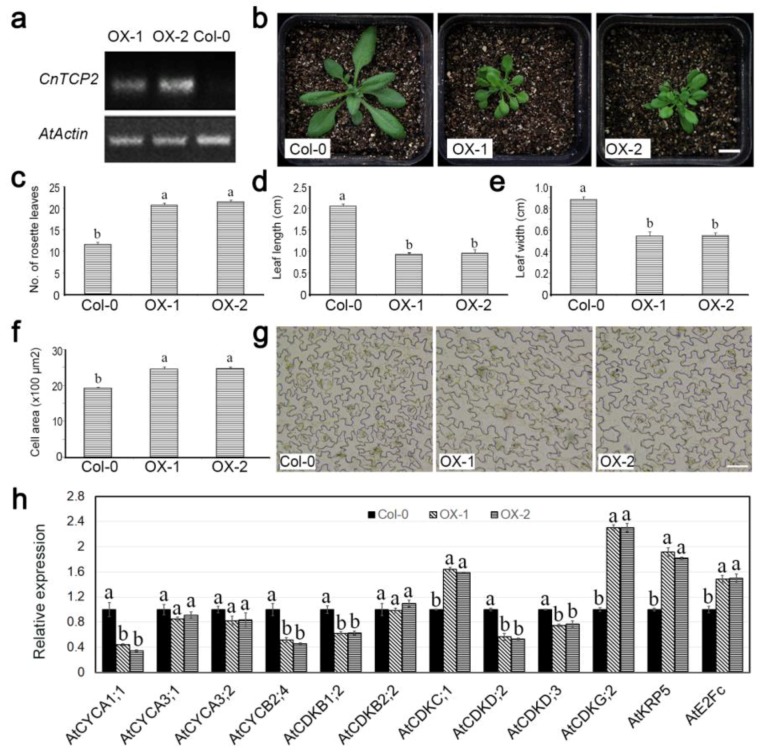
The phenotypic effect of constitutively expressing *CnTCP2* in *A. thaliana*. (**a**) RT-PCR-based identification of the transgenic *A. thaliana* plants OX-1 and OX-2. (**b**) The appearance of 35-day-old Col-0 wild type, and transgenic OX-1 and OX-2 plants. Bar: 1 cm. Quantification of leaf growth. (**c**) number of rosette leaves. (**d**) length of the fifth leaf. (**e**) width of the fifth leaf. (**f**) surface area of cells in the fifth leaf. Values in (**c**) through (**f**) shown as mean ± SE (*n* = 10. (**g**) The appearance of epidermal cells sampled from the fifth leaf. Bar: 50 µm. (**h**) The transcription of cell cycle marker genes. Col-0: wild type plants; OX-1, OX-2: transgenic plants. Values shown as mean ± SE *(n* = 3). Columns headed by a different letter denote transcript abundances differing significantly (*p* < 0.05) from the transcript abundance measured in Col-0 plants.

The full length *CnTCP2* cDNA sequence comprised 2066 nt, of which 1329 nt was occupied by the ORF: its gene product was 442 residues long. The sequence was classified as a class II TCP, related more strongly to *AtTCP2* than to any other *A. thaliana TCP* gene (Figure 1b). The sequence encoded an atypical bHLH domain (Figure 1c). The gene was abundantly transcribed in ligulate florets and leaves (Figure S2a), and was strongly downregulated in plants sprayed with 6-benzylaminopurine (Figure S2b). The gene product was directed to the nucleus (Figure S3a), and lacked transcriptional activation activity (Figure S3b). Analysis of the two independent constitutive expressors of *CnTCP2* in *A. thaliana* selected among the T_3_ progeny bred from the original transformed Col-0 material (Figure 9a) showed that that presence of the transgene affected growth in the same way as did the constitutive expression of *CnTCP4* (Figure 9b–g). The abundance of *CYCA1;1*, *CYCB2;4*, *CDKB1;2*, *CDKD;2,* and *CDKD;3* transcripts were lower in the transgenic plants than in Col-0, and vice versa for *CDKC;1*, *CDKG;2*, *KRP5,* and *E2Fc* (Figure 9h).

## 3. Discussion

TCP transcription factors, featuring a bHLH motif that facilitates their ability to bind to DNA and to mediate protein–protein interactions, are unique to plants, and contribute to both their growth and development [6]. Both the *CnTCP4* and *CnTCP2* genes harbored by *C. nankingense* encode class II TCPs. Proteins of this type have been implicated in the regulation of several aspects of plant development, including the development of axillary meristems and the differentiation of lateral organs [3,21,22]. *Antirrhinum majus cin* mutants (*CIN* encodes a TCP transcription factor that promotes growth arrest, particularly at the leaf margin) develop larger leaves than do wild-type plants, and their leaves have an undulating edge due to excessive growth at the leaf margin [23]. The *A. thaliana JAW* locus generates a microRNA, which guides the cleavage of several *TCP* mRNAs controlling leaf development, and its loss-of-function mutant exhibits a crinkly leaf phenotype [24]. The product of *AtTCP3* is thought to be an important regulator of shoot lateral organ morphology [21], while the expression of a hyperactivated form of *AtTCP4* dampens cell proliferation, producing plants with cup-shaped leaves of reduced size [25]. At the early stages of leaf development, the TIE1 protein has been shown to modulate leaf size and shape through its inhibition of TCP activity [26]. Here, it was demonstrated that the constitutive expression of *CnTCP4* in *A. thaliana* had the effect of reducing leaf size, indicating that its product represses leaf development.

CIN-like TCP transcription factors are known to regulate growth though their interaction with phytohormone-associated pathways. For instance, *AtTCP3* activates the auxin signaling repressor *IAA3/SHY2* [27]. The expression of the rice gene *OsTCP5* is influenced by both strigolactone and cytokinin [28]. Meanwhile AtTCP4 interacts with pathways associated with auxin, gibberellin, and abscisic acid [29]. It also promotes the synthesis of jasmonate via the upregulation of *LOX2* [11,30]. In the present study, ethylene-, gibberellin-, jasmonate- and salicylic-responsive regulators were all identified within the *CnTCP4* promoter sequence (Table 1), suggestive of the participation of CnTCP4 in the phytohormone-directed regulation of plant growth, a conclusion supported by the transcriptional responsiveness of *CnTCP4* to treatment with the synthetic cytokinin 6-benzylaminopurine (Figure 2). Cytokinin 6-benzylaminopurine promoted cell division by repressing the expression of *CnTCP4*. 

*AtTCP4* has been implicated in coordinating cell division and cell differentiation in the developing leaf; since the downregulation of *AtTCP4* has been shown to promote mitotic activity [24,31], the overexpression of this gene induces cells to exit the proliferation phase prematurely, resulting in the formation of smaller leaves [25]. Here, the constitutive expression of *CnTCP4* in fission yeast caused the cells to elongate and to become multiseptated, while also suppressing cell division; its constitutive expression in *A. thaliana* reduced leaf size and expanded the size of the epidermal cells on the abaxial leaf surface. It showed a significant increase in average epidermal cell size in *tcp20* mutant leaves, but no obvious size or shape alterations could be observed in the *tcp20* mutant line, owing to the fact that the cell size effect was accompanied by a reduction in total cell number in the leaf [11]. Smaller leaf phenotypes could be observed in overexpressed *CnTCP2/4* lines, indicating that the cell size effect is completely compensated for by a reduction of the total number of cells in the leaf. The suggestion is that these effects derive from the transgene product’s suppression of cell proliferation, allied with its promotion of cell expansion. This notion is fully consistent with the observation that the tetraploid form of *C. nankingense* formed larger leaves than did the diploid form, while the abundance of *CnTCP4* transcript was significantly lower (Figure 1b).

The timing of the onset of cell proliferation and expansion is correlated with the transition from cell division to the endocycle [32]. Cell division is orchestrated by a number of proteins, including cyclins, CDKs, and CDK inhibitors [33]. The activity of the CDK–cyclin complex is regulated by CDK-activating enzymes and CDK inhibitory proteins [34,35]. Here, all of *AtCYCA1;1*, *AtCYCA3;1*, *AtCYCA3;2*, *AtCYCB2;4*, *AtCDKB1;2*, *AtCDKB2;2*, *AtCDKD;2,* and *AtCDKD;3* were downregulated in plants constitutively expressing *CnTCP4*, which may explain the observed reduction in leaf size induced by the transgene, because each of these genes acts to suppress cell division. The CDKCs are responsible for the phosphorylation of the C terminal domain of RNA polymerase II. According to Kitsios et al. (2008), the function of the plant CDKC–CycT complex may be similar to that of the mammalian CDK9/CycT complex of the positive transcription elongation factor b [36]. In *A. thaliana,* a loss of CDKC;2 activity promotes cell division [37]. The *A. thaliana* genome harbors two genes encoding G-type CDKs, one which complexes with CYCL1 to delay entry into the cell cycle [38]. AtKRP5 influences cell cycle progression at both the G1/S and G2/M transitions by inhibiting the kinase activity of the CYCD2/CDKB complex and reconstituted CYCD2-associated kinases [39]. AtE2FC is a further protein that regulates entry into the G1/S transition [40]. Here, the upregulation of *AtCDKC;1*, *AtCDKG;2*, *AtKRP5,* and *AtE2Fc* induced by the constitutive expression of *CnTCP4* may have acted to inhibit cell division, thereby contributing to the reduction in leaf size observed in the transgenic plants.

BHLH proteins regulate their downstream targets by interacting with sequences within their promoter [41]. The AtTCP20 protein binds to the promoter of *AtTCP9*, thereby triggering its expression [11]. Based on the yeast one hybrid experiment and the transient expression assay in *N. benthamiana* leaves carried out here, the CnTCP2 protein was concluded as capable of binding to the *CnTCP4* promoter. The constitutive expression of *CnTCP2,* as was also the case for *CnTCP4,* suppressed cell division and promoted cell expansion, which suggests the presence in *C. nankingense* of a TCP2–TCP4 pathway. CnTCP2 had no transcription activity, but the constitutive expression of *CnTCP2* in *A. thaliana* also generated the same phenotype as *CnTCP4* (Figure 6 and Figure 9), it is possible that CnTCP2 binds to *AtTCP4* and hence produces small plants.

## 4. Materials and Methods

### 4.1. Plant Materials

The accessions of diploid and autotetraploid *C. nankingense* used here were maintained by Nanjing Agricultural University’s Chrysanthemum Germplasm Resource Preserving Centre. Plants were propagated by cutting and grown in a 1:1 (*v*/*v*) mixture of soil and vermiculite. Rooted seedlings were grown in a greenhouse maintained at 22 °C during the day and at a minimum of 15 °C during the night; the relative humidity ranged from 70% to 75%. *A. thaliana* (ecotype Col-0) plants were grown in a 1:1:1 (*v*/*v*/*v*) mixture of vermiculite, perlite, and soil under a 16 h photoperiod (80–100 μmol m^−2^ s^−1^ illumination), and a day/night temperature regime of 22 °C/18 °C.

### 4.2. Transcription Profiling of CnTCP4

Transcription profiling was performed using quantitative real-time PCR (qRT-PCR), based on the primer pair CnTCP4-QF/QR (sequences given in Table S1). Total RNA was extracted from *C. nankingense* tissue using the RNAiso reagent (TaKaRa, Tokyo, Japan) according to the manufacturer’s instructions. Each qRT-PCR assay was represented by three technical replicates, and each sample by three biological replicates. The *C. nankingense* EF1α gene was used as the reference (primer sequences given in Table S1). The reactions were initially denatured (95 °C/2 min), then cycled 40 times through 95 °C/15 s, 55 °C/15 s, 72 °C/20 s. Relative transcript abundances were calculated using the 2^−^^△△*C*t^ method [42].

### 4.3. Cloning and Sequencing of CnTCP4 cDNA

Diploid *C. nankingense* leaves were snap-frozen in liquid nitrogen, and total RNA isolated using the RNAiso reagent (TaKaRa, Tokyo, Japan), following the manufacturer’s instructions. The first cDNA strand was synthesized from 1 μg total RNA using M-MLV reverse transcriptase (TaKaRa) according to the manufacturer’s instructions. The primer pair CnTCP4-F/-R (sequences given in Table S1) was designed to amplify a fragment of *CnTCP4* based on a sequence represented in a transcriptome database [43]. The full length cDNA sequence was obtained by applying 5’- and 3’-RACE PCR. For the 3’-RACE reaction, the first cDNA strand was generated using the dT adaptor primer, following by a nested PCR based on the primer pairs CnTCP4-3’-F1/F2/F3 and the Adapter-R adaptor primer (sequences given in Table S1). For the 5’ reaction, the nested PCR used the primers AAP and AUAP provided with a 5’ RACE System kit v2.0 (Invitrogen, Carlsbad, CA, USA), along with the gene-specific primers CnTCP4-5’-F1/-F2/-F3 (sequences given in Table S1). The PCR products were purified using an AxyPrep DNA Gel Extraction kit (Axygen, Shanghai, China) and inserted into pMD19-T (TaKaRa) for sequencing. Finally, a gene-specific primer pair (CnTCP4-ORF-F/-R, sequences given in Table S1) was designed to amplify the gene’s complete open reading frame (ORF) sequence.

### 4.4. Phylogenetic Analysis

*A. thaliana* TCP sequences were downloaded from the Arabidopsis Transcription Factors database [44], and combined with the newly acquired *CnTCP4* sequence to perform a multiple alignment analysis based on ClustalW software [45]. The subsequent phylogenetic analysis was based on the neighbor-joining method, as implemented in MEGA v6 software [46]. The robustness of each dendrogram branch was estimated by means of a bootstrap analysis (1000 replicates).

### 4.5. Phytohormone Treatments

The diploid form of *C. nankingense* at the 6–8 leaf stage were chosen to assess the transcriptional response of *CnTCP4* to 5.0 µM 6-benzylaminopurine treatment [47]. Control plants were sprayed with distilled water. The leaves were sampled both before the first spray treatment, and then again after 1, 2, 4, 8, 12, and 24 h. Each treatment was replicated three times. The leaf samples were snap-frozen in liquid nitrogen and stored at −70 °C.

### 4.6. Subcellular Location of CnTCP4

The *CnTCP4* ORF sequence (lacking the stop codon) was amplified with the primer pair CnTCP4-pENTR1A-F/R (sequences given in Table S1) using a Phusion High-Fidelity PCR kit (New England Biolabs, MA, USA). Both the amplicon and the pENTR™ 1A vector (Invitrogen) were restricted with *Bam*HI and *Not*I, and the products ligated using T4 DNA ligase (TaKaRa). Following its validation by sequencing, the pENTR™1A-*CnTCP4* fusion product was recombined with pMDC43 using the LR Clonase™ II enzyme mix (Invitrogen) to form the construct *p35S::GFP-CnTCP4*. The *p35S::GFP-CnTCP4* transgene or an empty pMDC43 vector were transiently transformed into onion epidermal cells using a PDS-1000 particle accelerator (Bio-Rad, Hercules, CA, USA) according to the manufacturer’s instructions. The epidermal peels were incubated in the dark at 22 °C for 16 h on Murashige and Skoog (MS) medium [48]. The expression of GFP was observed by confocal laser microcopy (Leica SP2, Wetzlar, Germany).

### 4.7. Transactivation Activity Assay of CnTCP4

The transactivation activity of CnTCP4 was tested with a yeast one hybrid assay [48]. The three different C’-deletion variants of the CnTCP4 coding region (Figure 3c) were amplified using the primer pair CnTCP4-pENTR1A-F plus one of CnTCP4-pENTR1A-R1/-R2/-R3 (sequences given in Table S1). The resulting fragments were inserted into pENTR™1A via the *Bam*HI and *Not*I cloning sites. The resulting pENTR™1A-*CnTCP4*, pENTR™1A-*CnTCP4-F1*, pENTR™1A-*CnTCP4-F2,* and pENTR™1A-*CnTCP4-F3* fusions were subsequently recombined with pDEST-GBKT7 via an LR reaction (Invitrogen) to form the constructs pDEST-GBKT7-*CnTCP4*, pDEST-GBKT7-*CnTCP4-F1*, pDEST-GBKT7-*CnTCP4-F2,* and pDEST-GBKT7-*CnTCP4-F3*. Each of these four constructs, plus pCL1 (positive control) and pDEST-GBKT7 (negative control), were introduced individually into Y2H Gold yeast cells (Clontech, Mountain View, CA, USA) following the manufacturer’s protocol. Selection for transformants (except for those carrying pCL1) was carried out by culturing the cells on SD/-Trp medium, while the pCL1 transformants were cultured on SD/-Leu medium. All of the transformant cell lines were finally plated on SD/-His/-Ade medium containing 20 mg/mL X-α-Gal, and incubated at 30 °C.

### 4.8. Yeast Transformation, Growth, and Observation

The *CnTCP4* coding sequence was amplified using primer pair CnTCP4-*Xho*I-F/ CnTCP4-*Bam*HI-R (sequences given in Table S1) and the resulting amplicon inserted into pESPM via its *Xho*I and *Bam*HI cloning sites to form the construct pESPM-*CnTCP4*. This construct (and in parallel, as a control, an empty pESPM plasmid) was transformed into *Schizosaccharomyces pombe* Leu^-^ SPQ-01 using a lithium acetate-mediated method, as recommended in a commercial protocol (Clontech), and transformants were selected on Edinburgh minimal medium (EMM) agar plates containing thiamine held at 30 °C. Selected cells were then cultured in liquid EMM containing thiamine held at 30 °C up to the mid-exponential phase, after which they were flushed three times with EMM lacking thiamine to de-repress the *NMT1* promoter. The same transgenic mid-exponential cells were then streaked onto liquid EMM (either with or without thiamine) and incubated at 30 °C for 30 h, and cell densities were monitored by measuring the OD_600_. The same number of transformed mid-exponential yeast cells were also streaked onto solid EMM medium with or without thiamine and incubated at 30 °C for 60 h. *CnTCP4* transcription was monitored using an RT-PCR assay (primer pair CnTCP4-*Xho*I-F/CnTCP4-*Bam*HI-R, sequences given in Table S1), after total RNA had been isolated from the cells using a Fungi RNA kit (Hua Yue Yang, Beijing, China). The *S. pombe Tubulin* gene was used as the reference (primer sequences given in Table S1). Cells were stained in a 1 mg/mL solution of 4,6-diamidino-2-phenylindole (DAPI) and monitored by fluorescence microscopy (Zeiss Axioskop40, CarlZeiss, Jena, Germany). Mean cell lengths were compared using routines implemented in SPSS v17.0 software (SPSS Inc., Chicago, IL, USA).

### 4.9. A. thaliana Transformation and Transcriptional Profiling of Cell Cycle Marker Genes

The *p35S::GFP-CnTCP4* construct was introduced into *Agrobacterium tumefaciens* strain EHA105, and from thence into *A. thaliana* Col-0 using the floral dip method [49]. Transformants were selected by culturing on half strength MS medium agar plates containing 20 µg/mL hygromycin, and were advanced to the T_3_ generation. The zygosity of the transgene was identified using a RT-PCR assay based on the primer pair CnTCP4-ORF-F/R (sequences given in Table S1). The size of epidermis cells from the abaxial surface of the fifth leaf of ten independent 35-day-old T_3_ plants was measured from phase contrast micrographs. The transcriptional behavior of selected cell cycle marker genes was derived from qRT-PCR assays, with the *AtActin* gene chosen as the reference (primer sequences given in Table S1). RNA was extracted from the fifth leaves of 28-day-old wild-type and transgenic plants using the RNAiso reagent (TaKaRa) according to the manufacturer’s instructions.

### 4.10. Cloning of CnTCP4 Promoter

Genomic DNA was extracted from leaves of diploid *C. nankingense* using a modified CTAB method [50], and used as a PCR template (primed by CnTCP4-DNA-F/-R, sequences given in Table S1) to scan for the presence of introns in the coding sequence. The *CnTCP4* promoter was cloned from the genomic DNA using an LA PCR^TM^ in vitro Cloning kit (TaKaRa), according to the manufacturer’s instructions, using the primer pair promoter-AP/CnTCP4-pro-SP1 (sequences given in Table S1). A second nested round of PCR was performed using 1 μL of the initial PCR as the template and the primer pair promoter-AP/CnTCP4-pro-SP2 (sequences given in Table S1). The final nested PCR used 1 μL of the second PCR as the template and the primer pair promoter-AP/CnTCP4-pro-SP3 (sequences given in Table S1). Primer pair CnTCP4-pro-F/R (sequences given in Table S1) was then used to amplify the *CnTCP4*_pro_-1336 sequence. Functional elements of *CnTCP4* promoter were predicted by PLACE (http://www.dna.affrc.go.jp/PLACE/signalup.html).

### 4.11. Yeast One Hybrid Assay

The primer pair CnTCP4-PHIS-F/R (sequences given in Table S1) was used to amplify the *CnTCP4*_pro_-1336 sequence and the amplicon was inserted into the pHISi plasmid (Clontech), which contains the *HIS3* reporter gene and a minimal *HIS3* promoter, to generate the reporter construct pHISi-*CnTCP4*_pro_-1336. The recombined plasmid was linearized by digestion with *Afl*II, and the resulting fragment introduced into brewers’ yeast (*S. cerevisiae*) strain YM4271 (Clontech) following the manufacturer’s protocol. The cells were cultured on SD/-His/-Ura medium for the selection of cells harboring the reporter plasmid, and then cultured on SD/-His/-Ura medium containing different 3-amino-1,2,4-triazole (3-AT) concentrations to select optimal 3-AT concentration. The selected cells, along with a premade Y1H cDNA library, were transformed into *S. cerevisiae* strain YM4271, and transformants were selected on SD/-His/-Ura/-Leu medium containing 30 mM 3-AT.

A gene-specific primer pair (CnTCP2-ORF-F/-R, sequences given in Table S1) was designed to amplify the complete open reading frame sequence of *CnTCP2*. The *CnTCP2* ORF sequence was inserted into the yeast expression plasmid pDEST-GADT7 (Clontech) to generate the effector plasmid pDEST-GADT7-*CnTCP2* (CnTCP2-pENTR1A-F/R, sequences given in Table S1). The pHISi-*CnTCP4*_pro_-1336 reporter plasmid and the pDEST-GADT7-*CnTCP2* effector plasmid were transformed into *S. cerevisiae* strain YM4271, and the transformants selected by culturing on a SD/-His/-Ura medium. Selected transgenic cells were then cultured on SD/-His/-Ura/-Leu medium containing 30 mM 3-AT to monitor the activation of the *CnTCP4* promoter.

### 4.12. Transient Expression Assay in Nicotiana benthamiana Leaves

A transient assay was used in *N. benthamiana* leaves to investigate whether CnTCP2 can interact with the *CnTCP4* promoter in planta. The primer pair CnTCP4-LUC-F/-R (sequences given in Table S1) was used to amplify the *CnTCP4* promoter and the amplicon was ligated into pCAMBIA1381Z-Luc (Clontech) to generate the reporter construct pCAMBIA1381Z-Luc-*CnTCP4-*Promoter. Overnight culturezzs of *Agrobacterium tumefaciens* GV3101 harboring the reporter construct, the effector construct *p35S::GFP-CnTCP2,* or an empty P19 plasmid were suspended in infiltration buffer (2 mM NaH_2_PO_4_, 50 mM MES, 100 μM acetosyringone, and 0.5% Glc) in sufficient density to give an OD_600_ of 0.2. Leaves of 42-day-old *N. benthamiana* plants were spot-infiltrated with the transgenic *Ag. tumefaciens* cells, following the Wang et al. (2014) protocol [51]. The experiment group was performed with cultures of P19 plus 35S::CnTCP2 and pCAMBIA1381Z-Luc-*CnTCP4-*Promoter at a ratio of 1:1:1. The control group were performed with P19 plus GV3101 at a ratio of 1:2, P19 plus GV3101 and 35S::CnTCP2 at a ratio of 1:1:1, P19 plus GV3101 and pCAMBIA1381Z-Luc-*CnTCP4-*Promoter at a ratio of 1:1:1. Luciferase was imaged 48–96 h after infiltration, following the Walley et al. (2007) protocol [52].

## Figures and Tables

**Table 1:** An in silico analysis of the regulatory element content of the *CnTCP4* promoter.

Motif	No.	Distance from ATG (bp)	Function
CAT-box	1	1116	*cis*-acting regulatory element related to meristem expression
CCGTCC-box	3	662, 1154, 1330	*cis*-acting regulatory element related to meristem specific activation
CGTCA-box	1	686	*cis*-acting regulatory element involved in the MeJA-responsiveness
ERE	1	998	ethylene-responsive element
P-box	2	994, 1022	gibberellin-responsive element
TCA-element	1	32	*cis*-acting element involved in salicylic acid responsiveness
TGACG-box	1	686	*cis*-acting regulatory element involved in the MeJA-responsiveness
ARE	2	92, 159	*cis-*acting regulatory element essential for the anaerobic induction
EIRE	1	902	elicitor-responsive element
Box-W1	2	913, 957	fungal elicitor responsive element
Skn-1 motif	2	106, 681	*cis*-acting regulatory element required for endosperm expression
LTR	3	969, 1086, 1261	*cis*-acting element involved in low-temperature responsiveness
CAAT-box	2	1276, 1292	common *cis*-acting element in promoter and enhancer regions
MRE	1	1189	MYB binding site involved in light responsiveness
GT1-motif	1	1105	light responsive element
Sp1	1	826	light responsive element
Box I	4	353, 639, 994, 1168	light responsive element
AAAC-motif	1	412	light responsive element

## Supplementary Materials

Supplementary materials can be found at https://www.mdpi.com/1422-0067/20/19/4848/s1.
