# Supplementary material for: The Heterologous Expression of a Chrysanthemum nankingense TCP Transcription Factor Blocks Cell Division in Yeast and Arabidopsis thaliana
Source: Int J Mol Sci. 2019 Sep 29;20(19):4848. doi: 10.3390/ijms20194848 (PMC6801669; doi:10.3390/ijms20194848)
Supplement: Supplementary file 1 [file ijms-20-04848-s001.pdf]

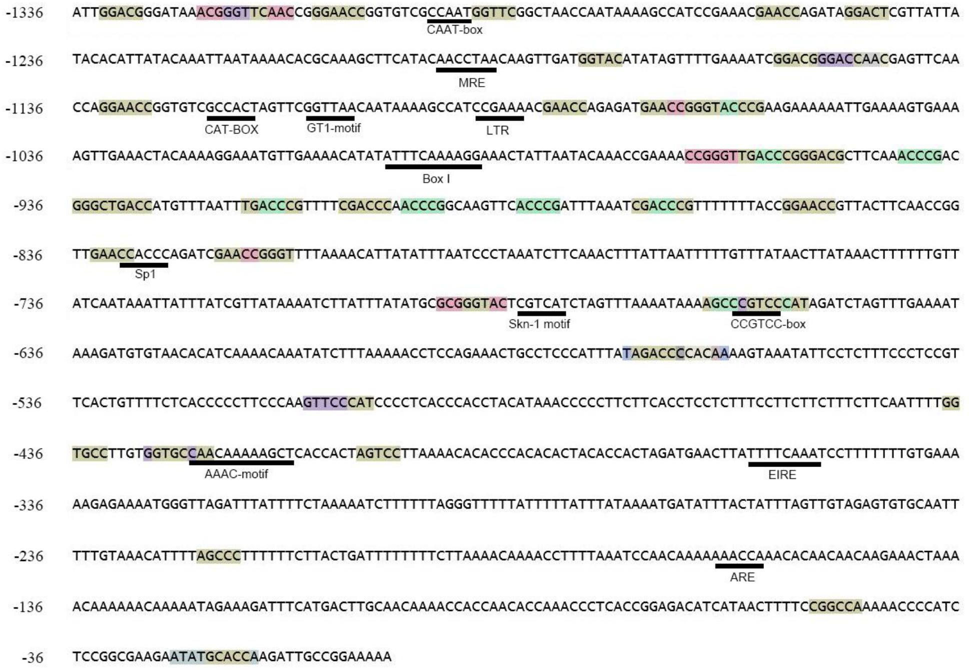

Figure S1. TCP binding sites and various elements present in the *CnTCP4* promoter. Putative TCP binding sites are shown in color. Different colors represented various TCP binding sites. Functional elements as predicted by PLACE software are underlined.

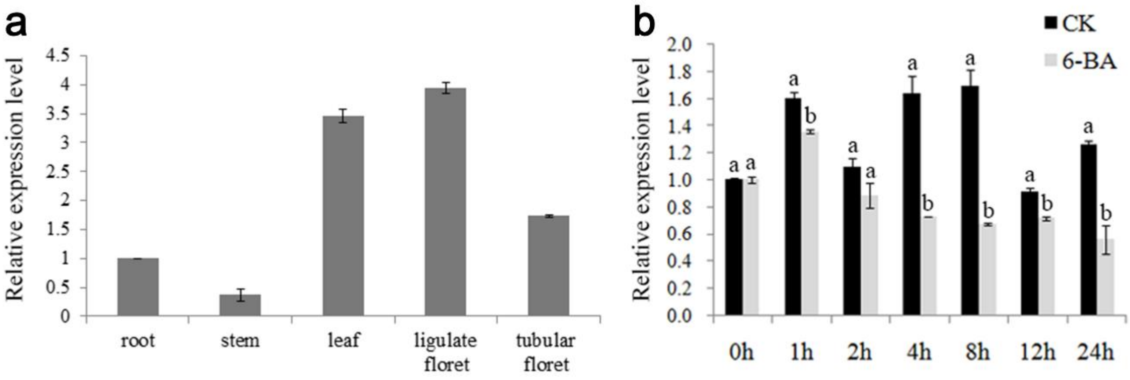

Figure S2. Transcriptional profiling of *CnTCP2*. (**a**) Variation in the transcript's abundance throughout the *C. nankingense* plant; (**b**) The effect of spraying the plant with 6- benzylaminopurine. Values shown as mean ±SE (*n*=3). Columns in (**b**) headed by a different letter denote transcript abundances differing significantly (P<0.05) from the transcript abundance measured in unsprayed plants.

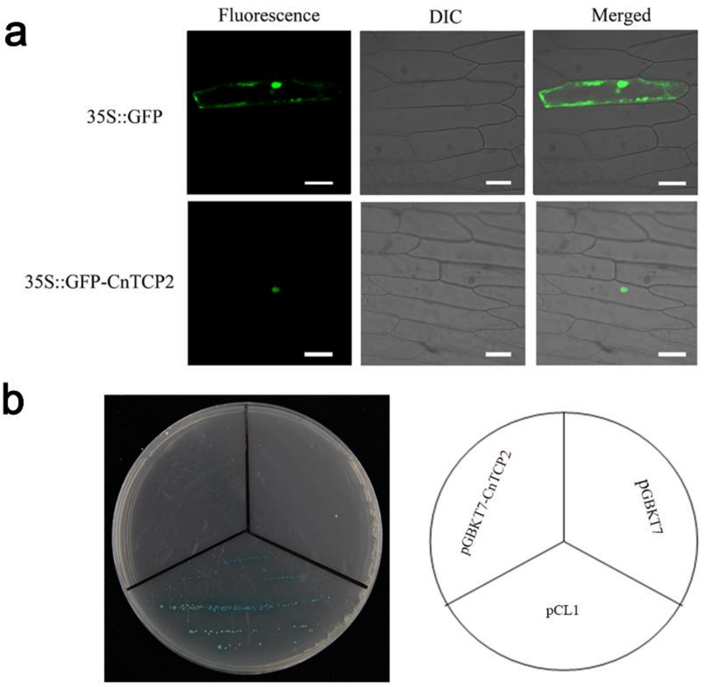

Figure S3. Subcellular localization of CnTCP2 in transgenic onion epidermal cells and the transcriptional activation activity of CnTCP2. (**a**) GFP activity generated by either a p*35S::GFP* or a p*35S::GFP-CnTCP2* transgene introduced into onion epidermis peels. Fluorescence: image recovered from the green fluorescence channel, DIC: image recovered under bright light, Merged: an overlay of the Fluorescence and DIC images. Bar: 50 μm; (**b**) A yeast one hybrid assay used to detect the transcriptional activation activity of CnTCP2.

Table S1. Primer sequences used in this study.

Primer	Sequence (5′-3′)
CnTCP4-F	GGGAATTGGAATTCAAGAG
CnTCP4-R	GGCCCATTCGAAGAAGCA
Oligo d(T)$_{18}$	GACTCGAGTCGACATCGATTTTTTTTTTTTTTTTT
Adapter	AAGCAGTGGTATCAACGCAGAGTACTTTTTTTTTTTTTTT
Adapter-R	AAGCAGTGGTATCAACGCAGAGTAC
CnTCP4-3′-F1	TGATGAAAATGGTAATGAAAGC
CnTCP4-3′-F2	AGTGGGAATGGGAATAGTAGTG
CnTCP4-3′-F3	CAGTTGTTTACTCAGAGGGGAC
AAP	GGCCACGCGTCGACTAGTACGGGIIGGGIIGGGIIG
AUAP	GGCCACGCGTCGACTAGTAC
CnTCP4-5′-F1	AAATGATTGAAGTGACAA
CnTCP4-5′-F2	AGAAGCACCCATAGGAAA
CnTCP4-5′-F3	ACTAGGTTTCCAAGCTG
CnTCP4-ORF-F	AACCACCAACACCAAACC
CnTCP4-ORF-R	GTCACATACATGGGCTAA
CnTCP4-QF	CAAGGTGATGATGAGGAGCA
CnTCP4-QR	ACTGTGTAGCAGCCATTGGA
CnEF1α-F	TTTTGGTATCTGGTCCTGGAG
CnEF1α-R	CCATTCAAGCGACAGACTCA
CnTCP4-pENTR1A-F	CGCGGATCCGGATGCCTGAAACATACCAA
CnTCP4-pENTR1A-R	AGTGCGGCCGCGAATGACGAGAATCCGAGGA
CnTCP4-pENTR1A-R1	AGTGCGGCCGCGACTGATCTGGGTCTTGCTC
CnTCP4-pENTR1A-R2	AGTGCGGCCGCGAATTAAACTGATGCATATC

CnTCP4-pENTR1A-R3	AGTGCGGCCGCGATGACATCTGCGGCGCCTG
CnTCP4-*Xho*I-F	CCGCTCGAGATGCCTGAAACATACCAA
CnTCP4-*Bam*HI-R	CGCGGATCCTCAATGACGAGAATCCGAGGA
Tubulin F	CAGCCAACTTTCTCAAATCAG
Tubulin R	GCCAAGGGTCACTACAC
CnTCP4-DNA-F	ATGCCTGAAACATACCAAT
CnTCP4-DNA-R	CCAATTCCCTGATCTGGG
promoter-AP1	NTCGASTWTSGWGTT
promoter-AP2	NGTCGASWGANAWGAA
promoter-AP3	WGTGNAGWANCANAGA
promoter-AP4	AGWGNAGWANCAWAGG
CnTCP4-pro-SP1	CAAGTTCATCAATGGCAGTTTTAG
CnTCP4-pro-SP2	GGTCTATCATAACCTAAACGGTCTTGT
CnTCP4-pro-SP3	TAAGGCTAATCTTGATGACCCTTG
CnTCP4-pro-F	ATTGGACGGGATAAACGG
CnTCP4-pro-R	GGTCTATCATAACCTAAACGGTCTTGT
CnTCP4-PHIS-F	CCGGAATTCATTGGACGGGATAAACGGG
CnTCP4-PHIS-R	TGCTCTAGATTTTTCCGGCAATCTTGGT
CnTCP4-LUC-F	CGCGGATCCATTGGACGGGATAAACGGG
CnTCP4-LUC-R	GCACTGCAGTTTTTCCGGCAATCTTGGT
CnTCP2-ORF-F	GCTTGGTATGGAGGTTGA
CnTCP2-ORF-R	CTGCCATCATAAATAGACAC
CnTCP2-pENTR1A-F	CGCGGATCCGGATGGAGGTTGATGAATTT
CnTCP2-pENTR1A-R	AGTGCGGCCGCGAGTTCTTTGATTTTTCCTT
AtCYCA1;1-F	AAAGCGATGGAGTTGAGAGG
AtCYCA1;1-R	GTGGGATTACGGATGGACAA
AtCYCA3;1-F	TCTTTCGGAGTTGAGCATGT
AtCYCA3;1-R	TTGGACGGATGATGAACCTA
AtCYCA3;2-F	CAAAGCGGCTGATCTACAAG
AtCYCA3;2-R	CAACGCATTGGAACTTATGG
AtCYCB2;4-F	GGCGATCTACACTGCTCAGA
AtCYCB2;4-R	TGATGCAAACCAACCATCTT
AtCDKB1;2-F	CTAGACCGCTTGAGGCTTCT
AtCDKB1;2-R	TTCTGCGGTTTAAGATCACG
AtCDKB2;2-F	GGTGAAGGGACTTATGGGAA
AtCDKB2;2-R	GCGAGCATACGCAAGATAGA
AtCDKC;1-F	GTTTCCAAGATGCCTTGGTT
AtCDKC;1-R	TGGATCAAGCACCAACATTT
AtCDKD;2-F	TCAAGCGTTTGGTACTCCAG
AtCDKD;2-R	AGCGTCATCACTTGCCATAG
AtCDKD;3-F	AGTGGCCGGATTTAACAAAG
AtCDKD;3-R	TTGGACAACAAGTCGAGAGC
AtCDKG;2-F	CCCGCTCAAGCCTTATACTC
AtCDKG;2-R	CATGATACAGCCCAGTGACC
AtKRP5-F	GCAGAAGCAACAGCTCATTC
AtKRP5-R	ATCCGGCTCTAATTTCTTCG
AtE2Fc-F	GGAAGGGTGCTGACAATCTT
AtE2Fc-R	GAGAGCTTCTTGTCGTTCCC
AtActin-F	GGTAACATTGTGCTCAGTGGTGG
AtActin-R	AACGACCTTAATCTTCATGCTGC
